# Cryo-EM structure of the human MON1A−CCZ1−RAB7A complex provides insights into nucleotide exchange mechanism

**DOI:** 10.1093/lifemeta/loaf017

**Published:** 2025-05-26

**Authors:** Xinna Li, Dan Li, Dan Tang, Xiaofang Huang, Hui Bao, Jiawei Wang, Shiqian Qi

**Affiliations:** Department of Urology, Institute of Urology, State Key Laboratory of Biotherapy, West China Hospital, College of Life Sciences, Sichuan University, and National Collaborative Innovation Center, Chengdu, Sichuan 610041, China; State Key Laboratory of Membrane Biology, Beijing Frontier Research Center for Biological Structure, School of Life Sciences, Tsinghua University, Beijing 100084, China; Department of Urology, Institute of Urology, State Key Laboratory of Biotherapy, West China Hospital, College of Life Sciences, Sichuan University, and National Collaborative Innovation Center, Chengdu, Sichuan 610041, China; Department of Urology, Institute of Urology, State Key Laboratory of Biotherapy, West China Hospital, College of Life Sciences, Sichuan University, and National Collaborative Innovation Center, Chengdu, Sichuan 610041, China; Department of Urology, Institute of Urology, State Key Laboratory of Biotherapy, West China Hospital, College of Life Sciences, Sichuan University, and National Collaborative Innovation Center, Chengdu, Sichuan 610041, China; State Key Laboratory of Membrane Biology, Beijing Frontier Research Center for Biological Structure, School of Life Sciences, Tsinghua University, Beijing 100084, China; Department of Urology, Institute of Urology, State Key Laboratory of Biotherapy, West China Hospital, College of Life Sciences, Sichuan University, and National Collaborative Innovation Center, Chengdu, Sichuan 610041, China

**Keywords:** RAB7A, MON1A−CCZ1, autophagy, nucleotide exchange mechanism, guanine nucleotide exchange factor, cryo-electron microscopy

## Abstract

Autophagy is a fundamental cellular process, conserved across species from yeast to mammals, that plays a crucial role in maintaining cellular homeostasis. The functionally conserved MON1−CCZ1 (MC1) complex serves as a guanine nucleotide exchange factor (GEF) for the RAB GTPase RAB7A and is indispensable for directing RAB7A recruitment to autophagosome or lysosomal membranes. Despite its critical role, the precise molecular mechanism underlying the assembly of the human MON1A−CCZ1 (HsMC1) complex and its specific GEF activity towards RAB7A has remained unclear. In this study, we report the high-resolution cryo-electron microscopy (cryo-EM) structure of the HsMC1 GEF domain in a complex with the nucleotide-free RAB7A^N125I^ at 2.85 Å resolution. Our structural data demonstrate that engagement with the HsMC1 complex induces marked conformational shifts in the phosphate-binding loop (P-loop) and Switch I/II regions of RAB7A. A striking feature of this complex is the direct interaction between the P-loop of RAB7A and CCZ1, a structural detail not previously observed. Furthermore, biochemical assays targeting residues within Interface I or II of the HsMC1−RAB7A complex highlight their critical role in mediating the interaction and suggest a unique mechanism for nucleotide exchange facilitated by the HsMC1 complex. These findings provide novel molecular insights into the functional mechanisms of the HsMC1−RAB7A complex, offering a robust structural framework to inform future investigations into disease-related targets and therapeutic development.

## Introduction

Autophagy, a highly conserved cellular process, maintains metabolic homeostasis through degrading and recycling of cytoplasmic components, thereby orchestrating energy balance and lipid metabolism regulation [1–6]. This lysosome-dependent pathway is intrinsically linked to the endomembrane system, an interconnected network of organelles that coordinates intracellular trafficking and compartmentalization through dynamic membrane remodeling [7]. Central to both systems are RAB GTPases, molecular switches within the Ras superfamily that govern vesicle dynamics via GTP/GDP cycling [8, 9]. These evolutionarily conserved proteins precisely control autophagosome biogenesis, maturation, and lysosomal fusion through spatiotemporal recruitment of effector complexes [10, 11]. Notably, dysfunction of RAB-mediated membrane trafficking perturbs autophagic flux, establishing direct mechanistic links to a broad spectrum of human pathologies, including neurodegenerative disorders, cancer, and metabolic diseases [12–15]. Emerging evidence highlights the pivotal role of RAB GTPases in metabolic regulation, wherein their dysregulation contributes to impaired insulin signaling [16], altered lipid homeostasis [17], and the pathogenesis of metabolic syndromes such as type 2 diabetes [18] and nonalcoholic fatty liver disease [19].

The spatiotemporal activation of RAB GTPases is precisely controlled by guanine nucleotide exchange factors (GEFs) that catalyze GDP−GTP exchange, and GTPase-activating proteins (GAPs) that accelerate GTP hydrolysis [20]. Notably, GEFs activate RAB GTPases and direct their membrane targeting through coordinated interactions [21]. This activation triggers conformational changes in RAB GTPases that are essential for their interaction with downstream effectors. Two conserved structural elements mediate these conformational transitions: the Switch I and Switch II regions, which undergo nucleotide-dependent reorientation [22, 23]. The phosphate-binding loop (P-loop) stabilizes nucleotide phosphates through coordination with Mg^2+^ and conserved lysine residues [24].

Among RAB family members, RAB7A serves as a master regulator of late endosomal maturation, lysosomal biogenesis, and autophagosome−lysosome fusion [25–28]. Its yeast ortholog, Ypt7, is activated by the Mon1−Ccz1 complex, a heterodimeric GEF containing three conserved longin domains (LDs) that mediate membrane association and RAB GTPase interaction [29, 30]. Previous studies suggest that membrane microenvironments enhance yeast Mon1−Ccz1 GEF activity [31, 32]. In metazoans, the MON1−CCZ1 (MC1) complexes have evolved through the incorporation of an additional non-tri-longin domain (non-TLD) subunit, RMC1 (Regulator of MON1−CCZ1) in humans and Bulli in *Drosophila*, suggesting functional specialization in higher eukaryotes [33, 34]. Activated RAB7 subsequently recruits effector complexes like HOPS (homotypic fusion and vacuole protein sorting) to mediate membrane tethering, while GAPs such as TBC1D5 (TBC1 domain family member 5) terminate signaling through GTP hydrolysis [35]. RAB7 activity is also required to maintain mitophagy, and a decrease in RAB7 leads to defects in mitochondrial autophagosome formation and the accumulation of damaged mitochondria [36, 37]. This regulatory axis is particularly critical for autophagy, where MON1−CCZ1 -dependent activation of RAB7 facilitates autophagosome maturation through ATG8-mediated recruitment [38, 39]. GORASP2 (Golgi reassembly stacking protein 2) regulates RAB7A by modulating MON1A−CCZ1, affecting its interaction with HOPS and promoting autophagosome maturation [40].

Previous structural studies have resolved the cryo-electron microscopy (cryo-EM) structure of fungal Mon1−Ccz1 complex and the crystal structure of their LD1 domain in complexes with nucleotide-free Ypt7 from *Chaetomium thermophilum* (Ct), providing valuable insights into the complex formation and GEF-mediated activation mechanisms in fungi [41, 42]^.^ More recently, cryo-EM structures of the MON1−CCZ1−Bulli complex from *Drosophila melanogaster* (Dm) have further advanced our understanding of the assembly and regulation of the MC1 complex in metazoans [43, 44]. Despite these insights, critical questions remain regarding the structural basis of RAB7A regulation in mammals, including the architecture of the human MON1−CCZ1−RAB7A complex, its divergence from yeast counterparts, the molecular determinants governing GEF−RAB specificity, and the mechanistic details of nucleotide displacement, particularly the role of metazoan-specific subunits in stabilizing the nucleotide-free state. Furthermore, while yeast structures provide foundational insights, the functional relevance of human-specific features cannot be extrapolated from fungal models.

To address these knowledge gaps, we present the high-resolution cryo-EM structure of the human MON1A−CCZ1−RAB7A^N125I^ (HsMCR7^N125I^) complex at 2.85 Å resolution, capturing RAB7A in a nucleotide-free state stabilized through unprecedented interactions with CCZ1. Our structural and mutational analyses reveal metazoan-specific regulatory features, including direct P-loop engagement by CCZ1 and interface-dependent control of nucleotide exchange. These findings establish a mechanistic framework for understanding RAB7A activation in human membrane trafficking pathways and provide critical insights for targeting autophagy-related disorders.

## Results

### Cryo-EM structure determination of the HsMCR7^N125I^ complex

To elucidate the structural architecture of the human MON1A−CCZ1 (HsMC1) complex, we first established a robust purification strategy for biochemical and structural studies. Although the low sequence identity (46.5%) between human MON1A and its paralog MON1B, AlphaFold 3 indicated that MON1A and MON1B exhibit near-identical structural topologies (Supplementary Fig. S1a and b). Hence, we focused on MON1A for further structural analysis.

**Figure 1:**
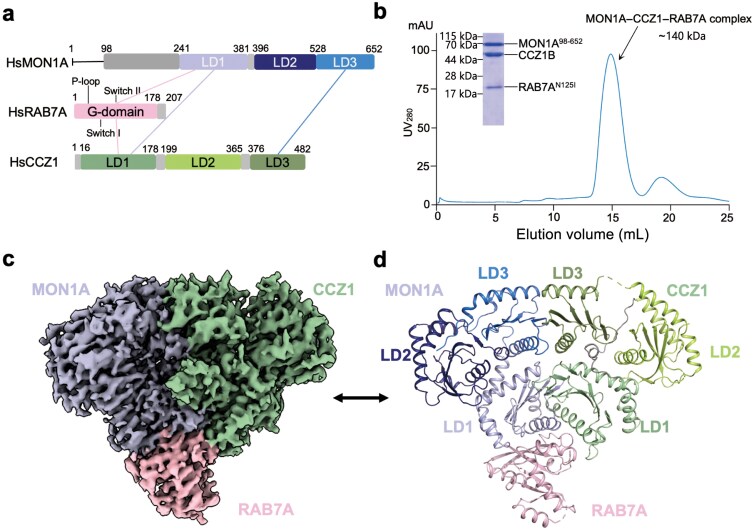
Cryo-EM structure of the HsMC1 complex bound to the nucleotide-free RAB7A^N125I^. (a) Schematic diagram of the domain arrangement in HsMON1A (blue), HsCCZ1 (green), and HsRAB7A (light pink). LD1 to LD3 and the G-domain (P-loop, Switch Ⅰ, and Switch Ⅱ) are indicated. Hs: *Homo sapiens*. The names and boundaries of domains are labeled. Interactions between domains are shown with colored lines: light blue for MON1A−CCZ1-LD1 interaction, marine blue line for MON1A−CCZ1-LD3 interaction, and light pink lines for RAB7A−MON1A or RAB7A−CCZ1 interaction. (b) Gel filtration (superpose 6 10/300 GL) profile of the reconstituted HsMCR7^N125I^ complex. The horizontal axis is elution volume, and the vertical axis is ultraviolet (UV) absorbance. The UV absorbance of the HsMCR7^N125I^ complex is shown in blue, with the protein peak labeled. A Coomassie blue-stained SDS-PAGE gel is shown for the peak fraction from gel filtration. (c) Cryo-EM map of the HsMCR7^N125I^ complex, with each subunit colored accordingly: light blue for MON1A, pale green for CCZ1, and light pink for RAB7A. (d) Cryo-EM model of the HsMCR7^N125I^ complex in cartoon representation. The MON1A domains (LD1−LD3) are colored in light blue, density blue, and marine blue, respectively; the CCZ1 domains (LD1−LD3) are colored in pale green, limon green, and smudge green, respectively; and RAB7A is shown in light pink.

Structural prediction revealed that the N-terminal region (residues 1−247) of HsMON1A is intrinsically disordered (Supplementary Fig. S1c). Moreover, residues 1−97 of HsMON1A display minimal cross-species conservation (Fig. 1a; Supplementary Fig. S2a), suggesting their exclusion from the GEF functional core. Hence, the disordered N-terminal region of MON1A was removed to facilitate the expression of the HsMC1 complex, and HsMON1A (98–652)−CCZ1, suitable for biophysical and biochemical analysis, was finally purified. Using this complex, we performed pull-down assays and confirmed that only RAB7A^T22N^ (the GDP-bound mutant), but not the RAB7A^WT^ or RAB7A^Q67L^ (the GTP-bound mutant), was able to associate with the HsMC1 complex (Supplementary Fig. S1d). Notably, the interaction between HsMC1 and RAB7A^T22N^ was relatively weak *in vitro*, prompting us to explore alternative strategies to obtain the stable complex. The Ypt7^N125I^ (nucleotide-free mutant of Ypt7) has been shown to better mimic the nucleotide exchange intermediate and to form more stable complexes with GEFs in previous studies [42], and therefore we attempted to utilize this variant. Due to the RAB7A^N125I^ (the nucleotide-free mutant) exhibiting poor solubility when expressed alone, we co-expressed HsRAB7A^N125I^ with HsMC1 in Sf9 insect cells via dual baculovirus infection, enabling efficient complex assembly (Fig. 1b).

We then carried out a single-particle cryo-EM analysis of the purified HsMCR7^N125I^ complex and yielded a three-dimensional (3D) cryo-EM reconstruction at an overall resolution of 2.85 Å in C1 symmetry (Fig. 1c; Supplementary Table S1). Local resolution refinement revealed well-defined density for key interaction interfaces, permitting *de novo* atomic model building with minimal reliance on homologous templates (Supplementary Fig. S3a−d). The final structure demonstrated that HsMC1 adopts a heterodimeric architecture, with MON1A and CCZ1 each comprising three tandem LDs (LD1−LD3). RAB7A engages the complex through its highly conserved G domain. Inter-subunit interactions within HsMC1 are mediated by β-sheet fusion between LD1 and LD3 domains of MON1A and CCZ1, forming two continuous β-sheets that stabilize the heterodimer. Intriguingly, flexible regions including the N-terminus of MON1A (residues 98−240), the CCZ1 N-terminal peptide (residues 1−15), and the C-terminal hypervariable region of RAB7A (residues 179−207) were unresolved due to conformational dynamics (Fig. 1a and d).

### Structural delineation of the HsMC1–RAB7A interaction interface

**Figure 2:**
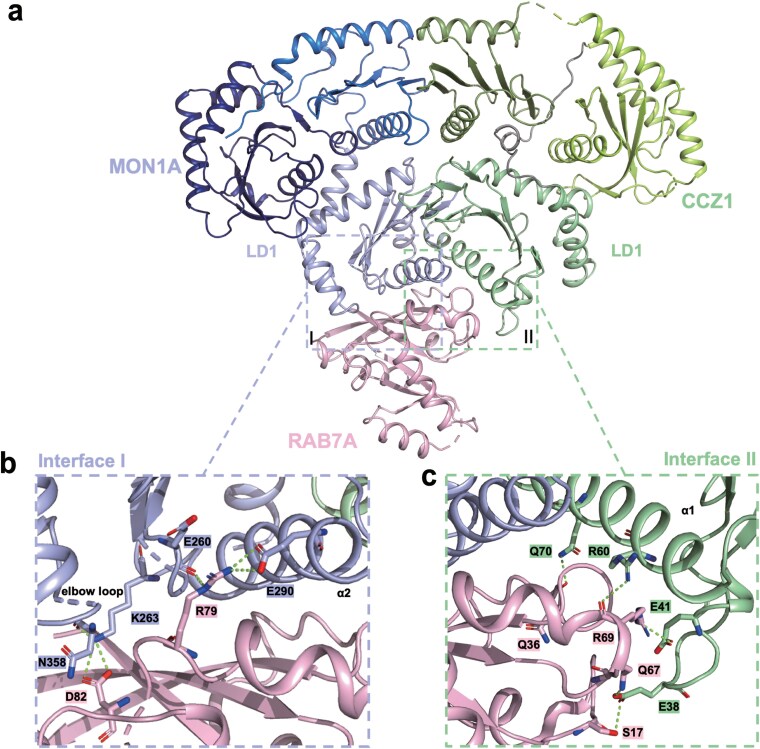
The interfaces of the HsMC1 complex and RAB7A^N125I^. (a) Cartoon representation showing the overall structure of the HsMCR7^N125I^ complex, colored as in Fig. 1d. (b) Cartoon-stick representation showing the interface I between MON1A and RAB7A^N125I^. (c) Cartoon-stick representation showing the interface II between CCZ1 and RAB7A^N125I^. Key residues at the interfaces are shown in the stick model, colored according to the chain color. Secondary structures are depicted as cartoons, and hydrogen bonds are indicated as green dotted lines.

Consistent with evolutionary conservation of GEF−RAB interfaces, the primary RAB7A binding sites are located within the LD1 domain of HsMC1, with MON1A contributing predominantly and CCZ1 playing a minor role (Fig. 2a). High-resolution density maps resolved key residues at the HsMC1−RAB7A interfaces (Supplementary Fig. S4a−c), involving RAB7A’s nucleotide-sensitive regions: the Switch I (N30−D44), Switch II (A65−G80), and the P-loop (G15−T22) [45].

The HsMC1−RAB7A interface is stabilized by a bipartite network of hydrogen bonds and hydrophobic packing. MON1A engages RAB7A through polar contacts between its E260/E290 side chains and RAB7A R79, as well as K263 and RAB7A D82 (Fig. 2b). Notably, HsMON1A K263 corresponds to CtMON1 K233, both of which interact with D82 of HsRAB7A and CtYpt7, respectively [42]. Sequence alignments indicate that E260 serves as a metazoan-specific interaction node absent in fungal systems, while E290 is conserved only in humans and Ct (Fig. 2a; Supplementary Fig. S2a). CCZ1 contributes complementary interactions via hydrogen bonds between E41/R60 and RAB7A R69, Q70 and RAB7A Q36, and E38 and RAB7A Q67/S17 (Fig. 2c). Among these, only CCZ1 Q70 aligns positionally with CtCCZ1 E113 [42], suggesting its role in stabilizing the interface through lineage-specific remodeling. Evolutionary analysis highlights that E41 serves as a metazoan-specific interaction node absent in fungal systems, E38 is conserved with variation only in CtCCZ1, while R60 is conserved in both HsCCZ1 and CtCCZ1 but is not retained in ScCCZ1 or DmCCZ1 (Supplementary Fig. S5a).

Two α-helical elements, MON1A α2 (S276−E290) and CCZ1 α1 (K55−T72), form the scaffold of the LD1 interaction platform. Intriguingly, MON1A harbors a conserved “elbow loop” (L350−N364) containing the metazoan-specific NYDLR motif (Fig. 2a; Supplementary Fig. S2a). Although this loop lacks direct RAB7A contacts, N358 forms an intramolecular hydrogen bond with K263, potentially rigidifying the adjacent interaction interface (Fig. 2b). In contrast, CCZ1’s analogous loop (N31−K44) directly engages RAB7A via E38/E41-mediated polar interactions, positioning the loop proximal to the RAB7A surface (Fig. 2c). Our structure resolves longstanding questions regarding the divergence of mammalian RAB7A regulation. The identification of non-conserved interfacial residues and lineage-specific motifs (NYDLR) underscores adaptive specialization in higher eukaryotes, demonstrating that metazoan tri-longin GEFs employ both conserved and novel interfacial strategies to achieve high-fidelity RAB7A activation.

### Evolutionary divergence of the HsMCR7^N125I^ complex architecture

**Figure 3:**
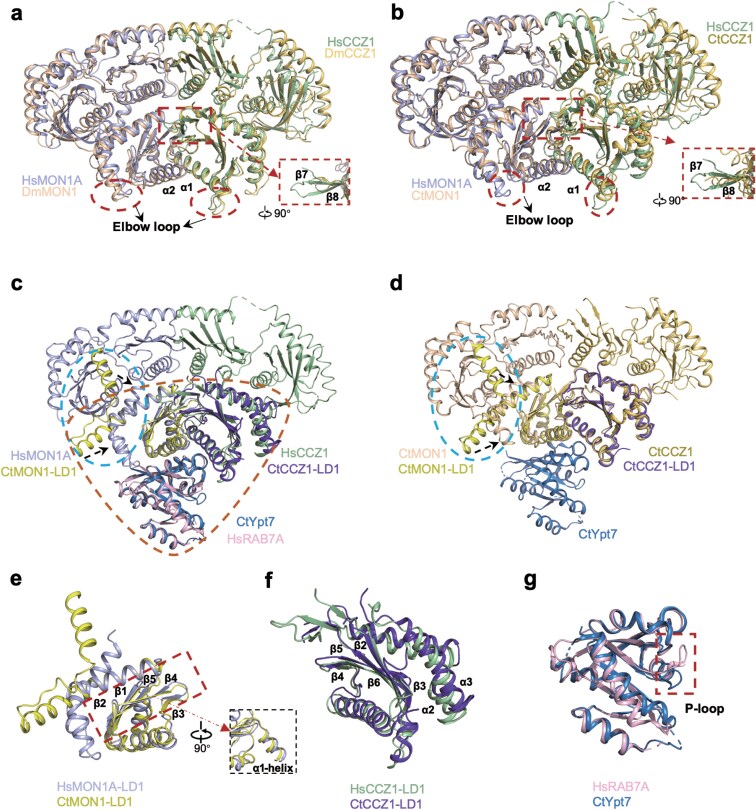
Structural comparison of HsMCR7^N125I^ across different species. (a and b) Superposition of HsMC1 with the DmMC1 subcomplex from the MON1−CCZ1−Bulli complex (PDB ID: 8C7G) (a) and the CtMC1 complex (PDB ID: 7QLA) (b). (c and d) Structural comparison of the CtMON1−CCZ1−Ypt7 core (PDB ID: 5LDD) with the HsMCR7^N125I^ complex (c) and the CtMC1 complex (PDB ID: 7QLA) (d). The blue dashed circles highlight the corrected structural region. (e and f) Zoomed-in view of the HsMON1A LD1 (e) and HsCCZ1 LD1 (f) domains compared with CtMC1. (g) Zoomed-in view of RAB7A^N125I^ compared with CtYpt7^N125I^. Panels (e−g) are derived from the structural alignment shown in panel (c), with red dashed circles resembling triangular shapes. Subunits of the HsMCR7^N125I^ complex are colored as in Fig. 1c. Secondary structures of the HsMC1^N125I^ complex are labeled as indicated, with distinct regions highlighted by red dotted boxes.

To delineate structural adaptations across evolutionary lineages, we performed a comparative analysis of the HsMC1 complex with its fungal (CtMC1) [41] and insect (DmMC1−Bulli) [43, 44] counterparts. Despite low sequence conservation (Hs versus Ct: MON1A 29.71%, CCZ1 20.86%; Hs versus Dm: MON1A 40.12%, CCZ1 29.85%; Supplementary Figs. S2b and S5b), all three complexes retain a conserved tri-longin domain (TLD) core architecture. Apart from the overall structural similarity, notable structural changes were identified in the LD1 domains of CtMC1 and DmMC1 that are distinct from those in HsMC1. In DmMC1−Bulli, the α2-helix of MON1 and α1-helix of CCZ1 adopt a downward shift relative to HsMC1, while the “elbow loop” of HsMC1 LD1 exhibits a pronounced inward curvature absent in CtMC1 and the β-strands (β7−β8) in HsCCZ1 are longer compared to those in DmCCZ1 and CtCCZ1 (Fig. 3a and b).

We then focused on the interface of MON1−CCZ1 with RAB7A/Ypt7. Furthermore, the core LD1 of HsMC1−RAB7A is also conserved and can be aligned with the CtMC1−Ypt7 [42] (PDB: 5LDD) structure. These conformational shifts correlate with species-specific loop dynamics: the CtMC1−Ypt7 structure lacks resolved density for its LD1 C-terminal loop (residues 314−353), precluding observation of fungal “elbow loop”−Ypt7 interactions (Fig. 3c and d). We also observed an additional α1-helix protruding protein surface at the N-terminal of HsMON1A (Fig. 3e). The HsMON1A LD1 α1-helix (E241−L250) is homologous to the CtMON1 α1-helix (D211−G220) (Supplementary Fig. S2a). While the LD1−RAB7A/Ypt7 interface is topologically conserved, metazoan complexes exhibit distinct interaction strategies. Certain regions of HsMC1 exhibit slight angular differences, with the β1−β5 of MON1A, and the β2−β6 and α2−α3 helices of CCZ1 displaying subtle variations when compared to CtMC1 (Fig. 3e and f).

The LD2 and LD3 domains play a crucial role in membrane association, particularly those containing phosphatidylinositol phosphate ( PIP) lipids, and are required for proper localization of the protein to endosomal structures [46]. Notably, human-specific loop deletions in LD2−LD3 suggest evolutionary streamlining of non-essential regulatory elements (Supplementary Figs. S2a and S5a). The functional domain sequence of RAB7A/Ypt7 is conserved across different species (Supplementary Fig. S6a and b), and a key distinction in RAB7A lies in its P-loop architecture, with structural differences between HsRAB7A and CtYpt7 primarily involving P-loop conformation (Fig. 3g). This structural shift correlates with the unique CCZ1-mediated P-loop engagement observed in HsMC1 as we previously described, a feature absent in fungal systems.

These analyses highlight a paradigm of conserved TLD scaffolding coupled with lineage-specific interfacial adaptations. The human complex employs distinct loop conformations, helix reorientations, and β-sheet extensions to establish a metazoan-optimized RAB7A interface. Such structural plasticity likely underlies functional specialization in higher eukaryotes, enabling nuanced regulation of autophagy and endosomal maturation.

### Identification of critical interaction residues in the HsMCR7^N125I^ complex

**Figure 4:**
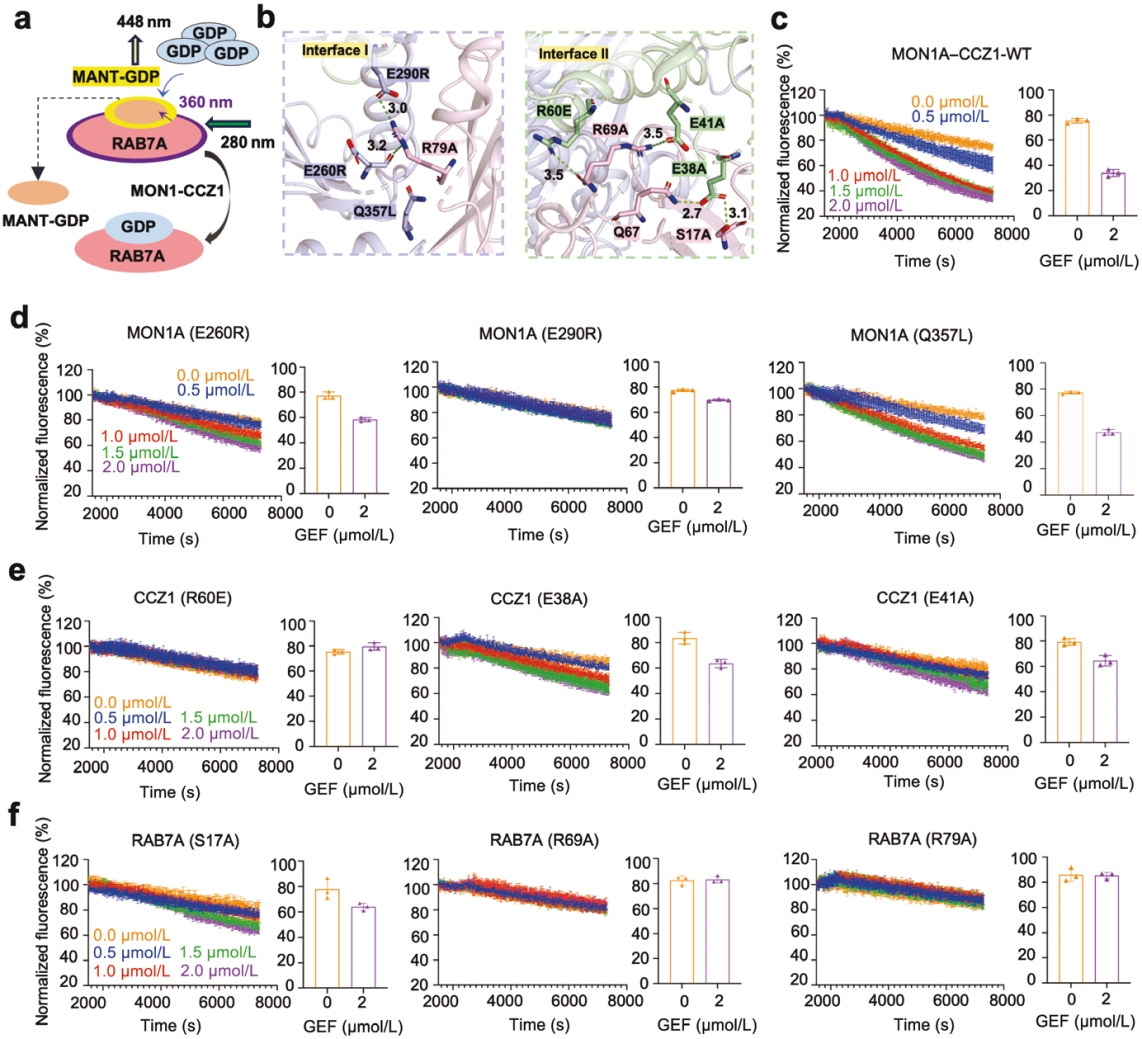
Identification of key residues critical for the interaction between HsMC1 and RAB7A. (a) Schematic diagram of GEF activation assays using fluorescent MANT-GDP in solution. The assay monitors nucleotide exchange via the fluorescence properties of MANT-GDP. Tryptophan residues in RAB7A are excited at 280 nm, producing fluorescence at 360 nm, which in turn excites MANT-GDP bound to RAB7A, allowing fluorescence detection at 448 nm. Dissociated MANT-GDP does not emit detectable fluorescence, enabling differentiation between bound and unbound states to assess GEF activity. (b) Structural details of the RAB7A interfaces. Left: Interface I with MON1A. Right: Interface II with CCZ1. Mutated residues are depicted as stick models. (c) MANT-GDP release monitored in the presence of RAB7A-WT and the WT HsMC1 complex. (d) MANT-GDP release monitored in the presence of RAB7A-WT and the HsMC1 complex with different MON1A mutants (E260R, E290R, and Q357L). (e) MANT-GDP release monitored in the presence of RAB7A-WT and the HsMC1 complex with different CCZ1 mutants (E38A, E41A, and R60E). (f) MANT-GDP release monitored with RAB7A mutants (S17A, R69A, and R79A) and the WT HsMC1 compex. Reactions were triggered by adding 100 μmol/L GDP. Fluorescence measurements were carried out every 15 s for a total of 7200 s. The right panels represent the quantification of fluorescence values at endpoint from the measurements in the left, in which the highest concentration of the HsMC1 complex was used to compare with the control. Error bars represent SD (*n* = 3). The plots are from at least three independent experiments.

To validate the structural determinants of HsMC1−RAB7A^N125I^ interaction, we employed a fluorescence resonance energy transfer (FRET)-based GEF assay [47, 48]. In cells, the typical cytosolic ratio of GTP to GDP is around 10:1, which is important for GTPase cycling and nucleotide exchange reactions [49]. Recombinant RAB7A (wild-type (WT) or mutants) preloaded with the fluorescent GDP analog N-methylanthraniloyl GDP (MANT-GDP) was incubated with titrated HsMC1, 10-fold molar excess of GDP was used as the competitor of MANT-GDP. Nucleotide exchange levels were quantified by monitoring the fluorescence change resulting from MANT-GDP displacement (Fig. 4a).

Structural and sequence analyses indicated that the LD1 domains of MON1A and CCZ1 are essential for GEF activity. As an initial exploratory strategy, we generated triple mutants MON1A^E260R/E290R/Q357L^ and CCZ1^E38R/E41R/R60E^. These mutants completely abolished GEF activity in the solution-based assay compared to WT HsMC1 (Supplementary Fig. S7a–c). To dissect residue-specific contributions, we then generated and purified a series of single mutants, including MON1A^E260R^, MON1A^E290R^, MON1A^Q357L^, CCZ1^E38A^, CCZ1^E41A^, CCZ1^R60E^, as well as RAB7A mutants RAB7A^R17A^, RAB7A^R69A^, and RAB7A^R79A^ (Fig. 4b). The mutants were well expressed and successfully purified. Subsequent GEF assays identified E290 in MON1A and R60 in CCZ1 as critical residues, resulting in a nearly complete loss of HsMC1 GEF activity. The CCZ1^E38A^, CCZ1^E41A^, MON1A^Q357L^, and MON1A^E260R^ mutants significantly reduced GEF activity towards RAB7A compared to WT HsMC1 (Fig. 4c–e; Supplementary Fig. S7c−e). As for RAB7A, RAB7A^R69A^ and RAB7A^R79A^ showed almost no response to HsMC1 stimulation, while RAB7A^S17A^ showed significant response to HsMC1 (Fig. 4f; Supplementary Fig. S7c and f).

Prior studies emphasized MON1A’s “elbow loop” function in GEF activity [42], and we identified the CCZ1 N31−K44 loop as equally critical (Figs. 2c and 4e). Notably, we identified Ser17 in the P-loop of RAB7A as a key residue in interaction (Figs. 2c and 4f).

### Structural basis of HsMC1-mediated nucleotide exchange mechanism for RAB7A

**Figure 5:**
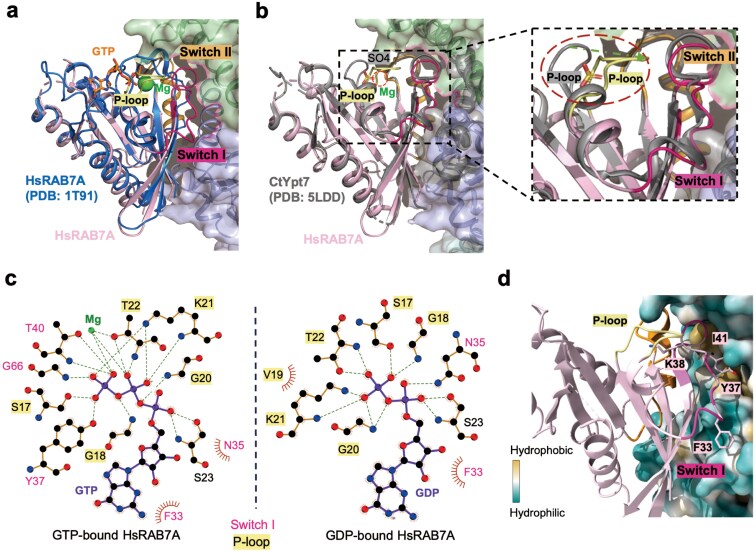
Structure insights into the mechanism of HsMC1. (a) Superposition of HsRAB7A^N125I^ (colored in light pink) bound to the HsMC1 complex with GTP-bound HsRAB7A (PDB: 1T91, colored in blue). Mg^2+^ in the GTP-bound HsRAB7A is shown as green spheres. Switch I (N35 displacement: 19.7 Å), Switch II (L73 shift: 6.3 Å), and the P-loop (G20 movement: 10.7 Å) undergo substantial conformational shifts. (b) Superposition of HsRAB7A^N125I^ (colored in light pink) bound to the HsMC1 complex with CtYpt7 (colored in gray) subunit from the CtMC1−Ypt7 complex (PDB: 5LDD). The location of the proposed Mg^2+^ is indicated by a red dashed line. The right panel presents a close-up view of HsRAB7A and CtYpt7 (gray), with the P-loop region highlighted within a red dashed circle. (c) Schematic representation of interactions formed between GTP and HsRAB7A (PDB: 1T91) (left) and between GDP and HsRAB7A (PDB: 8ZQ3) (right). (d) Interaction of Switch I in HsRAB7A with hydrophobic pockets on the HsMC1 complex. Residues F33, Y37, K38, and I41 are displayed as sticks and labeled. The surface of the HsMC1 complex is colored according to hydrophobicity (green for hydrophilic regions and yellow for hydrophobic regions). The P-loop, Switch I, and Switch II of HsRAB7A^N125I^ are colored in pale yellow, hot pink, and orange, respectively.

Comparative analysis of GTP-bound and HsMC1-bound RAB7A revealed large-scale rearrangements in nucleotide-sensitive regions: Switch I (19.7 Å), Switch II (6.3 Å), and P-loop (10.7 Å) (Fig. 5a). Unlike fungal CtMC1−Ypt7 complexes where sulfate ions might occupy the β-phosphate position, our cryo-EM structure captured RAB7A in an ion-free intermediate state. Here, the P-loop region of RAB7A undergoes a conformational shift towards the Mg^2+^-binding site (Fig. 5a and b), strongly indicating the critical role of P-loop in nucleotide exchange. Further interaction analysis between nucleotides and RAB7A also suggests that P-loop is pivotal for RAB7A’s interaction with nucleotides, regardless of GDP or GTP binding (Fig. 5c).

It has been reported that K41 in RAB11 positions itself towards the vacant Mg^2+^-binding site when bound to its GEF SH3BP5, with primary binding residues at the interface forming a contiguous hydrophobic surface [50]. Similarly, when HsMC1 binds to nucleotide-free RAB7A, Switch I disengages from the nucleotide, and K38 of RAB7A inserts into the Mg^2+^-binding pocket. In the GDP-bound state, F33, Y37, and I41 become buried, no longer exposed to the solvent (Supplementary Fig. S8a−c). The hydrophobic interactions between HsMC1 and RAB7A, involving Switch I residues (F33, Y37, and I41) and Switch II residues (R69, L73, and R79), further destabilize nucleotide binding by restricting solvent access (Fig. 5d; Supplementary Fig. S8d and e).

**Figure 6:**
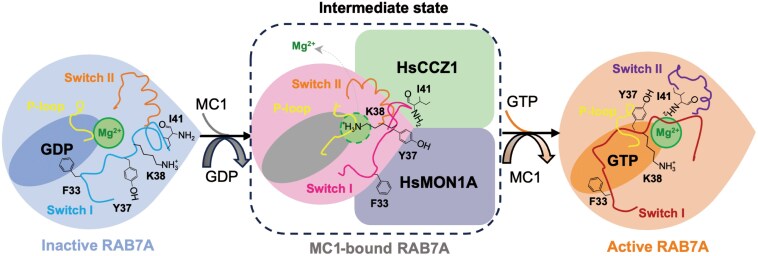
Model of nucleotide exchange mechanism by the HsMC1 complex. In the GDP-bound state, Switch I directly interacts with the nucleotide, with F33 stabilizing this interaction. Upon binding to the HsMC1 complex, Switch I undergoes a significant conformational rearrangement, where F33, Y37, and I41 of Switch I establish hydrophobic contacts with the HsMC1 complex, leading to the exposure of the nucleotide-binding pocket. The K38 residue projects into the Mg^2+^-binding pocket and the P-loop is also closer to the Mg^2+^-binding site. There are minimal conformational changes in Switch II when binding to the HsMC1 complex. Upon GTP binding, the Switch I and P-loop of RAB7A revert to their original conformations, while Switch II adopts a less helical and more relaxed structure. The P-loop, Switch I, and Switch II of HsRAB7A are colored in pale yellow, hot pink, and orange, respectively.

Despite conserved Switch I/II remodeling across species, metazoan RAB7A exhibits unique regulatory features. The P-loop’s directional shift in RAB7A^N125I^ contrasts with static conformations in fungal Ypt7^N125I^ (Fig. 5b; Supplementary Fig. S8f). The P-loop is critical for nucleotide interaction, a feature that is also highly conserved in yeast (Fig. 5c; Supplementary Fig. S8g). While the core functional regions of RAB7A/Ypt7 are highly conserved, the amino acid at position 35 in Switch I remains unchanged across metazoans but differs in fungi (Supplementary Fig. S6a), presumably suggesting adaptive optimization for mammalian membrane trafficking. Our data support a two-step nucleotide displacement model: MON1A-mediated Switch I restructure weakens GDP binding, while CCZ1-driven P-loop rearrangement disrupts Mg^2+^ coordination, enabling passive nucleotide exchange (Fig. 6). This cooperative mechanism distinguishes HsMC1 from fungal Mon1−Ccz1, which relies solely on Switch region remodeling (Fig. 5a; Supplementary Fig. S8f).

## Discussion

This study resolves the high-resolution cryo-EM structure of the HsMCR7^N125I^ complex, capturing RAB7A in a nucleotide-free state (Fig. 1d). The findings establish LD1 helices in both MON1A and CCZ1 as non-redundant functional modules, with evolutionary divergence in residue conservation dictating species-specific interaction strategies (Supplementary Figs. S2a and S5a). A hallmark discovery is a direct interaction between CCZ1-LD1 and RAB7A’s P-loop, a feature unobserved in fungal Mon1–Ccz1 complexes (Fig. 2c). To our knowledge, this represents the structural evidence of a TLD GEF directly modulating the P-loop architecture of a RAB GTPase, suggesting a metazoan-specific mechanism for nucleotide displacement. Notably, LDs are involved not only in GEF activity but also in GAP function, as seen in certain complexes. For instance, in the C9orf72–SMCR8–WDR41 complex, both C9orf72 and SMCR8 contain LDs that form a heterodimeric GAP core essential for RAB GTPase regulation [51, 52].

Our data reveal that the LD1 domains of MON1A and CCZ1 collaboratively remodel RAB7A’s nucleotide-binding pocket through three coordinated actions, including MON1A-driven displacement of Switch I to expel Mg^2+^; CCZ1-mediated P-loop reorientation to destabilize GDP binding, and remodeling of Switch II to stabilize the GEF complex (Fig. 5a and c). Integrating these observations with existing RAB7A structural states (Supplementary Fig. S8a−c), we propose a sequential activation model (Fig. 6), wherein HsMC1 binds to RAB7A in an intermediate conformational state and changes its conformation for subsequent nucleotide exchange. This process activates RAB7A, enabling it to bind and recruit downstream effectors. Revealing the structural changes of RAB7A, particularly the previously unrecognized role of the P-loop in RAB7A, is crucial for understanding the activation mechanism of RAB GTPase and its role in membrane trafficking.

While prior studies emphasized Switch I/II remodeling as the primary GEF mechanism [53], such as the Rabin8−RAB8A complex [54] and Sec2p/Sec4p complex [55], our work identified direct P-loop engagement as a metazoan innovation. The interaction of CCZ1^E38^−RAB7A^S17^ induces a conformational change in the P-loop (Figs. 4b, 4e, 4f, and 5b), a mechanism absent in CtYpt7 activation. This finding highlights CCZ1 as an active participant in nucleotide displacement.

Here, we determined the cryo-EM structure of HsMCR7^N125I^ at the resolution of 2.85 Å. While this manuscript was in preparation, the cryo-EM structure of the human MON1A−CCZ1−C18orf8 (RMC1)−RAB7A^T22N^ complex was reported [56]. That study also examined interface mutations between MON1A−CCZ1−RMC1 and RAB7A^T22N^. However, our GEF assays identified several key residues that had not been previously reported, including S17 of RAB7A, E290 of MON1A, and R60 of CCZ1.

A structural comparison of the two complexes (Supplementary Fig. S9a) reveals that the LD1 domains responsible for RAB7A binding remain structurally conserved, with no significant alterations at the interface despite the presence of RMC1. This suggests that RMC1 does not directly affect the structural basis of MON1A−CCZ1-mediated RAB7A regulation. Further analysis of the RAB7A^T22N^ structure and our RAB7A^N125I^ structure indicates that the Switch II region is largely unchanged (Supplementary Fig. S9b). Notably, the RAB7A^T22N^ structure did not reveal any conformational changes in the P-loop region. In contrast, the directions of the amino acids F33, Y37, and I41 in Switch I exhibit deviations from those observed in our structure. These differences likely arise from the T22N mutation in the MON1A−CCZ1−RMC1−RAB7A^T22N^ complex, which traps RAB7A in an inactive conformation distinct from the intermediate state (RAB7A^N125I^) captured in our structure, which is closer to a physiological state.

Our structure reveals an interaction between Q67 of RAB7A and CCZ1 E38, indicating that RAB GTPases employ distinct pathways for GDP release. The glutamate in the Switch II region plays distinct roles in protein activation, with its function varying across different RAB GTPases and their associated regulatory processes [22]. The P-loop is a conserved sequence motif present in nucleotide-binding proteins, undergoing conformational changes during the nucleotide exchange process [48, 57]. Recent studies reveal that the P-loop exhibits dynamic conformational changes during nucleotide binding in non-RAB GTPases, such as protein kinases, where its rearrangement in the ADP-bound state modulates the overall conformation and enzymatic function of the complex [58].

The structural plasticity of the HsMC1−RAB7A interfaces holds broad biomedical relevance. MON1A−CCZ1 dysfunction contributes to Alzheimer’s disease by impairing autophagosome maturation [59], while CCZ1 depletion significantly reduces SARS-CoV-2 infection, highlighting its essential role in the endosomal entry pathway [60]. Furthermore, the structural divergence between MON1A and MON1B (Supplementary Fig. S1b) suggests isoform-specific regulatory roles. Both MON1A and MON1B participate in the regulation of endosomal maturation through their interaction with CCZ1 and subsequent activation of RAB7 [61], while only MON1B plays a particularly critical role in the docking phase of early endosome (EE) fusion [62]. As a core component of the MC1 complex, MON1A is essential for autophagy, particularly by regulating RAB7A localization and stability during mitophagy [37]. In cancer, both MON1B and CCZ1 are implicated in the progression of colorectal and cervical tumors, promoting cell proliferation, migration, and invasion [63, 64].

Furthermore, Charcot-Marie-Tooth type 2B (CMT2B) disease-associated RAB7A mutations cause hyperactive GTP-RAB7A, disrupting endolysosomal trafficking, autophagy, and lysosomal function [65–67]. Unraveling these mechanisms highlights RAB7 regulation as a therapeutic target to restore homeostasis in CMT2B and related neurodegenerative disorders. In this context, our findings that residues S17, R69, and R79 in RAB7A are critical for the interaction with the HsMC1 complex suggest that small molecules or peptides targeting these key interface residues could be explored to modulate HsMC1 GEF activity. Such modulators may offer therapeutic potential in diseases, such as Alzheimer’s disease and cancer, where dysregulation of the HsMC1−RAB7A axis has been implicated.

In conclusion, our study confirms that HsMC1 functions as a GEF for RAB7A and reveals that nucleotide exchange is accompanied by critical conformational changes in the P-loop, a structural rearrangement that has not been reported before. The interaction between HsMC1 and RAB7A destabilizes the nucleotide-binding pocket, facilitating GDP release and enabling GTP loading. Given the essential role of RAB7A in membrane trafficking, our results provide a mechanistic investigation for understanding how HsMC1 regulates RAB7A activation and its pathological disruptions.

### Limitations of the study

This study focuses on the structural and biochemical characterization of the human MON1A−CCZ1−RAB7A complex. However, the role of cellular context, such as membrane association, RAB5 interaction, and post-translational modifications, was not taken into consideration. Whether the mechanism observed *in vitro* fully reflects the *in vivo* process remains unclear. Moreover, the RMC1 subunit, which may contribute to complex stability or regulation, was not included in the structure. The absence of RMC1 limits our understanding of the complete GEF machinery. These questions will be addressed in future studies.

## Materials and methods

### Plasmid construction

DNA sequences encoding WT and mutant human MON1A and CCZ1 were subcloned into pFastBac Dual vector under the control of the p10 and the polyhedron promoters, respectively. Briefly, HsMON1A-(residues 98−652), carrying an N-terminal Strep-tag II, and HsCCZ1-FL (residues 1−482), carrying an N-terminal 8 × His-tag, were cloned into constructs, in which the tags could be removed by tobacco etch virus (TEV) protease. DNA encoding full-length RAB7A^N125I^ was subcloned into the pFastBac Dual vector under the control of the polyhedron promoter with an N-terminal 8 × His-tag which was also removable by TEV protease.

cDNA of RAB7A (1−207) was subcloned into pHisparallel2 with an N-terminal His_6_-tag that can be removed by TEV protease. Substitution mutations in RAB7A and MON1A−CCZ1, as well as the deletion of N-terminal residues of MON1A, were generated by overlap extension PCR [68]. The primers were designed using sdm-primer-v1.1Python program [69]. The mutant constructs were verified by DNA sequencing.

### Protein expression and purification

Sf9 cells were infected with baculoviruses encoding MON1A (98−652)−CCZ1(1−482) and RAB7A ^N125I^ at 1:1 with the bac-to-bac system (Life Technologies). The cells were infected and grown in medium (SinoBiological-MSF1) at 27°C and finally harvested after 72 h. Cells were pelleted by centrifugation at 2000 *g* for 15 min. The pellets were lysed in 25 mmol/L Tris-HCl, pH 8.0, 150 mmol/L NaCl, 0.5 mmol/L TCEP-HCl, 1 mmol/L PMSF, 0.8 µmol/L aprotinin, 1 µmol/L pepstatin, and 10 µmol/L leupeptin by French press. The lysate was then centrifuged at 17000 rpm for 40 min at 4°C.

The supernatants of HsMCR7^N125I^ complex were loaded onto Strep-Tactin resin (IBA Lifesciences) at 4°C and eluted with 50 mmol/L Tris-HCl, pH 8.0, 150 mmol/L NaCl, 0.5 mmol/L TCEP-HCl, and 50 mmol/L D-Biotin, pH 8.0. The eluate of targeted proteins was purified in a Superose 6 10/300 GL column (GE Healthcare) equilibrated with buffer I (25 mmol/L Tris-HCl, pH 8.0, 150 mmol/L NaCl, and 0.5 mmol/L TCEP-HCl). Peak fractions were pooled and digested with TEV protease at 4°C overnight. TEV, His-tag, and Strep-tag were removed by loading the solution onto Ni-NTA resin. Target proteins were further purified in a Superose 6 10/300 GL column equilibrated with buffer I. The peak fractions were pooled, and flash-frozen in liquid N_2_ for storage. The peak fractions were used for cryo-EM. All the WT and mutant HsMC1 complex were purified as the HsMCR7 complex, without removing tags. The proteins were stored in buffer II (25 mmol/L Tris-HCl, pH 8.0, 150 mmol/L NaCl, and 2 mmol/L DTT) for nucleotide exchange assay.

RAB7A-WT (1−207) was expressed in *Escherichia coli* BL21 (DE3) cells grown in LB medium at 37°C until the culture reached an OD_600_ of 0.8−1.0, followed by induction with 0.2 mmol/L IPTG (isopropyl-β-d-thiogalactopyranoside) at 16°C for 12−16 h. Cells were harvested by centrifugation at 4000 *g* for 15 min. The pellets were lysed in 25 mmol/L Tris-HCl, pH 8.0, 150 mmol/L NaCl, 0.5 mmol/L TCEP-HCl, and 1 mmol/L PMSF by French press. The lysate was then centrifuged at 17000 rpm for 30 min at 4°C. The supernatants of RAB7A were loaded onto Ni-NTA resin (QIAGEN) at 4°C; target proteins were eluted with 25 mmol/L Tris-HCl, pH 8.0, 150 mmol/L NaCl, 0.5 mmol/L TCEP-HCl, and 250 mmol/L Imidazole, pH 8.0; and the eluates were diluted 5-fold with 25 mmol/L Tris-HCl, pH 8.0 and applied to a Hi Trap Q HP column. Peak fractions were pooled and digested with TEV protease at 4°C overnight. TEV and His_6_-tag were removed by loading the solution onto Ni-NTA. Target proteins were further purified on a Superdex 75 increase 10/300 GL column equilibrated with buffer III (25 mmol/L Tris-HCl, pH 8.0, 150 mmol/L NaCl, 2 mmol/L MgCl_2_, and 2 mmol/L DTT). The peak fractions were pooled and flash-frozen in liquid N_2_ for storage.

All HsMC1 and RAB7A mutants exhibited comparable yields and chromatographic profiles to the WT proteins. Gel filtration chromatography confirmed that all mutants existed in a monomeric state.

### Pull-down assay

For the pull-down assay, the indicated versions of Strep-tagged MON1A−CCZ1 (15 μmol/L) and untagged-RAB7A (30 μmol/L) were incubated with Strep-Tactin resin in incubation buffer (150 mmol/L NaCl, 25 mmol/L Tris, pH 8.0, 0.05% NP40, and 5% glycerol) at 4°C for 40 min. After washing with incubation buffer for three times, the resin was resuspended in 1 × SDS loading buffer, boiled at 95°C for 5 min, and applied to SDS-PAGE analysis.

### Cryo-EM sample preparation and data collection

For negative staining, an aliquot of 4 μL sample containing purified HsMON1A−CCZ1−RAB7A complex was applied on a carbon film grid (Beijing XXBR Technology Co. Ltd.) after plasma glow-discharge. The grid was stained with uranyl acetate (2%, w/v) and stored at room temperature. The negatively stained sample was imaged on a Tecnai Spirit Bio TWIN microscope (Thermo Fisher) operating at 120 kV to verify the sample quality.

Cryo-EM sample was prepared with Vitrobot Mark IV (Thermo Fisher Scientific) by applying 4 μL of protein at 0.42 mg/mL to a glow-discharged holey gold grid (Quantifoil, R1.2/1.3 μmol/L, 300 mesh). The grid was gently blotted for 3.5 s at 8°C under 100% humidity, followed by a 30-s wait. The quality of the prepared grid was verified with a Tecnai Arctica 200 kV electron microscope equipped with a K2 camera (Gatan Company). Cryo-EM images were collected from assessed samples by a 300 kV Titan Krios electron microscope (Thermo Fisher Scientific) outfitted with a Falcon4 camera (Thermo Fisher Scientific). The image dataset was collected with a pixel size of 1.036 Å, a total dose of 50 e^−^/Å^2^, and 32 frames. The defocus of all micrographs was set to range from −1.5 to −2.5 µm. Data collection was fully automated and facilitated by AutoEMation software and EPU (Thermo Fisher Scientific).

### Cryo-EM data processing

The EM data processing procedure for the HsMON1A–CCZ1–RAB7A^N125I^ complex is outlined in Supplementary Fig. S3. A total of 6,406 motion-corrected micrographs were collected, and the contrast transfer function was initially re-estimated using cryoSPARC [70]. From these, 4,554,171 particles were initially picked using the blob picker, and after multiple rounds of 2D classification, 1,246,669 particles were selected as high-quality classes. These particles were extracted with a bin4 box size of 64 and used to generate several rounds of ab-initio reconstruction in C1 symmetry. Finally, after homogeneous refinement and postprocessing, 492,272 particles were re-extracted with a bin1 box size, and a map was generated at a resolution of 2.85 Å.

### Protein model building and structure refinement

The protein model was constructed *de novo* using EMBuilder [71]. Manual modifications to the models were conducted in COOT [72], followed by real-space structure refinement using PHENIX [73]. Statistical details of the 3D reconstruction and model refinement processes are provided in Supplementary Table S1. Visual representations of the structures were generated using UCSF ChimeraX [74, 75] and PyMol [76].

### AlphaFold modeling

The model of human MON1B was generated using AlphaFold3 on the AlphaFold Server (https://alphafoldserver.com/about) [77]. For the MON1B model, the sequence of MON1B (residues 1−547) was input into the query sequence module.

### Nucleotide exchange assay

The nucleotide exchange assay for HsMC1 was performed similarly to that of C9ORF72−SMCR8−WDR41 [52]. Purified RAB7A was loaded with MANT-GDP (Thermo Fisher, M12414) in the presence of 5 mmol/L EDTA, pH 8.0 and 2-fold molar excess of MANT-GDP at 4°C overnight. The loading reaction was terminated by adding MgCl_2_ to 20 mmol/L and the RAB7A−MANT-GDP complex was further purified using Superdex 75 increase 10/300 GL column in buffer III. For the nucleotide exchange assay, 2.5 μmol/L RAB7A−MANT-GDP complex was pre-incubated with 0, 0.5, 1.0, 1.5, and 2.0 μmol/L of HsMC1 complex for 1200 s. After baseline stabilization, the nucleotide exchange reaction was triggered by the addition of GDP to a final concentration of 100 μmol/L [42, 78]. The release of MANT-GDP was recorded by monitoring the decrease in fluorescence emission at 448 nm (excitation: 280 nm) in intervals of 15 s at 25°C for 6000 s, with a total assay duration of 7200 s. The fluorescence intensity at 25 min was normalized to 100% in all assays. Data were collected using a 384-well plate (Corning, 3701) in Bio Tek SynergyH1. Subsequent data analysis was performed employing Prism 9 software.

## Supplementary Material

loaf017_suppl_Supplementary_Figures_S1-S9_Table_S1

## Data Availability

All data supporting the findings of this study are available from the corresponding author upon request. The cryo-EM map and the structure have been deposited to the Electron Microscopy Data Bank (EMD-63249) and the Protein Data Bank (PDB: 9LOL), respectively. All relevant data are included in the main text and Supplementary Information files.
