# Supplementary material for: Cryo-EM structure of the human MON1A−CCZ1−RAB7A complex provides insights into nucleotide exchange mechanism
Source: Life Metab. 2025 May 26;4(5):loaf017. doi: 10.1093/lifemeta/loaf017 (PMC12448474; doi:10.1093/lifemeta/loaf017)
Supplement: loaf017_suppl_Supplementary_Figures_S1-S9_Table_S1 [file loaf017_suppl_supplementary_figures_s1-s9_table_s1.docx]

Supplementary material for

**Cryo-EM structure of the human MON1A-CCZ1-RAB7A complex provides insights into nucleotide exchange mechanism**

Xinna Li^1,‡^, Dan Li^2,‡^, Dan Tang^1,‡^, Xiaofang Huang^1^, Hui Bao^1^, Jiawei Wang^2,*^, Shiqian Qi^1,*^

^1^Department of Urology, Institute of Urology, State Key Laboratory of Biotherapy, West China Hospital, College of Life Sciences, Sichuan University, and National Collaborative Innovation Center, Chengdu, Sichuan 610041, China

^2^State Key Laboratory of Membrane Biology, Beijing Frontier Research Center for Biological Structure, School of Life Sciences, Tsinghua University, Beijing 100084, China

**
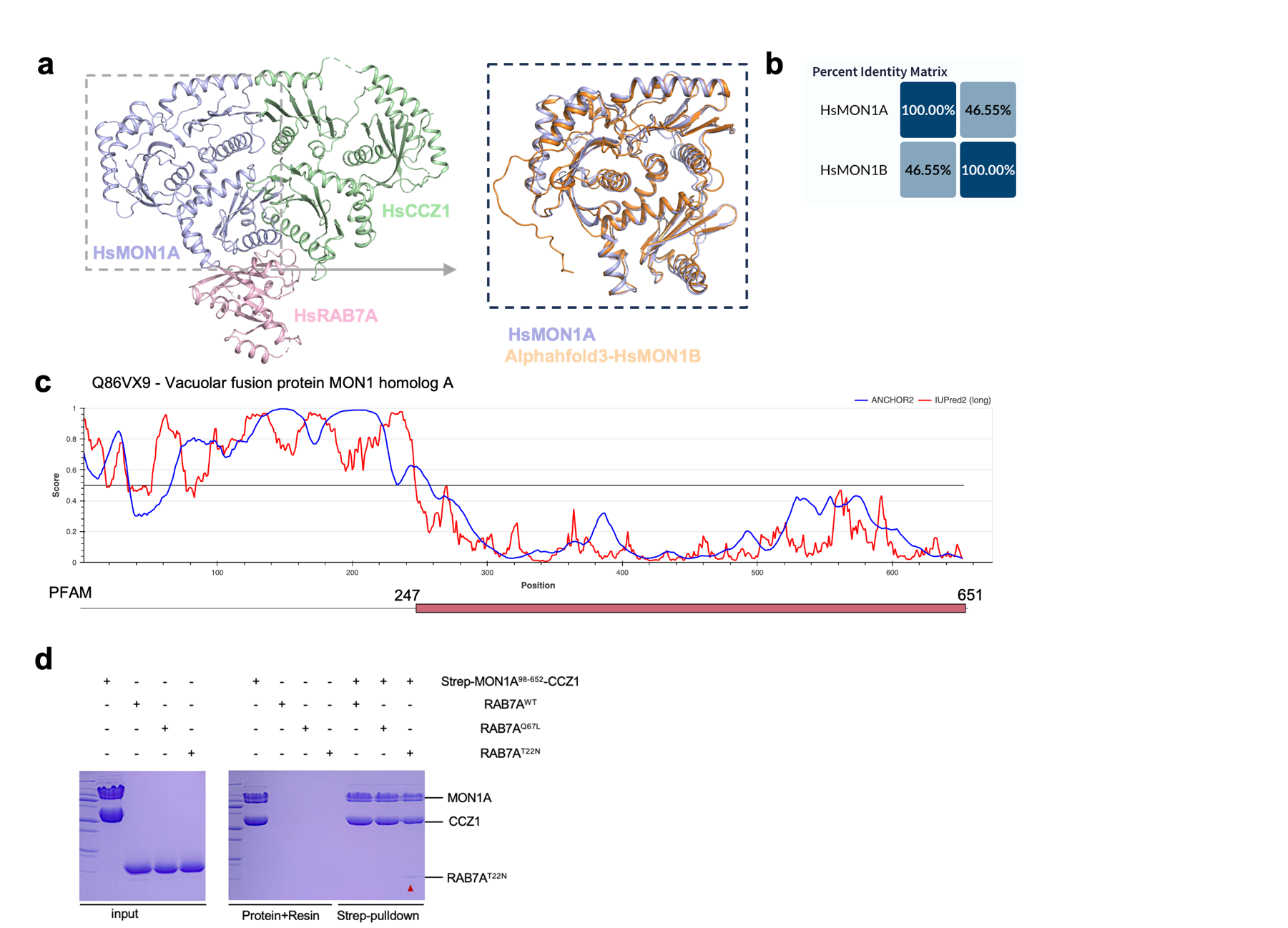
**

**Supplementary Figure S1** Structural analysis of MON1 and interaction analysis between the MON1A-CCZ1 complex and RAB7A. (a) Structural comparison between HsMON1A and HsMON1B (residues 101−547), with the predicted structure of HsMON1B generated using AlphaFold 3. (b) Sequence alignment of MON1A and MON1B in *Homo sapiens* (Hs). (c) The output of IUPred2 and ANCHOR2 predictions for HsMON1A, with IUPred2 scores in red and ANCHOR2 scores in blue. (d) Pull-down assay using Strep-tagged MON1A-CCZ1 to capture different forms of untagged RAB7A, including RAB7A wild-type (RAB7A^WT^), active form (RAB7A^Q67L^), and inactive form (RAB7A^T22N^).


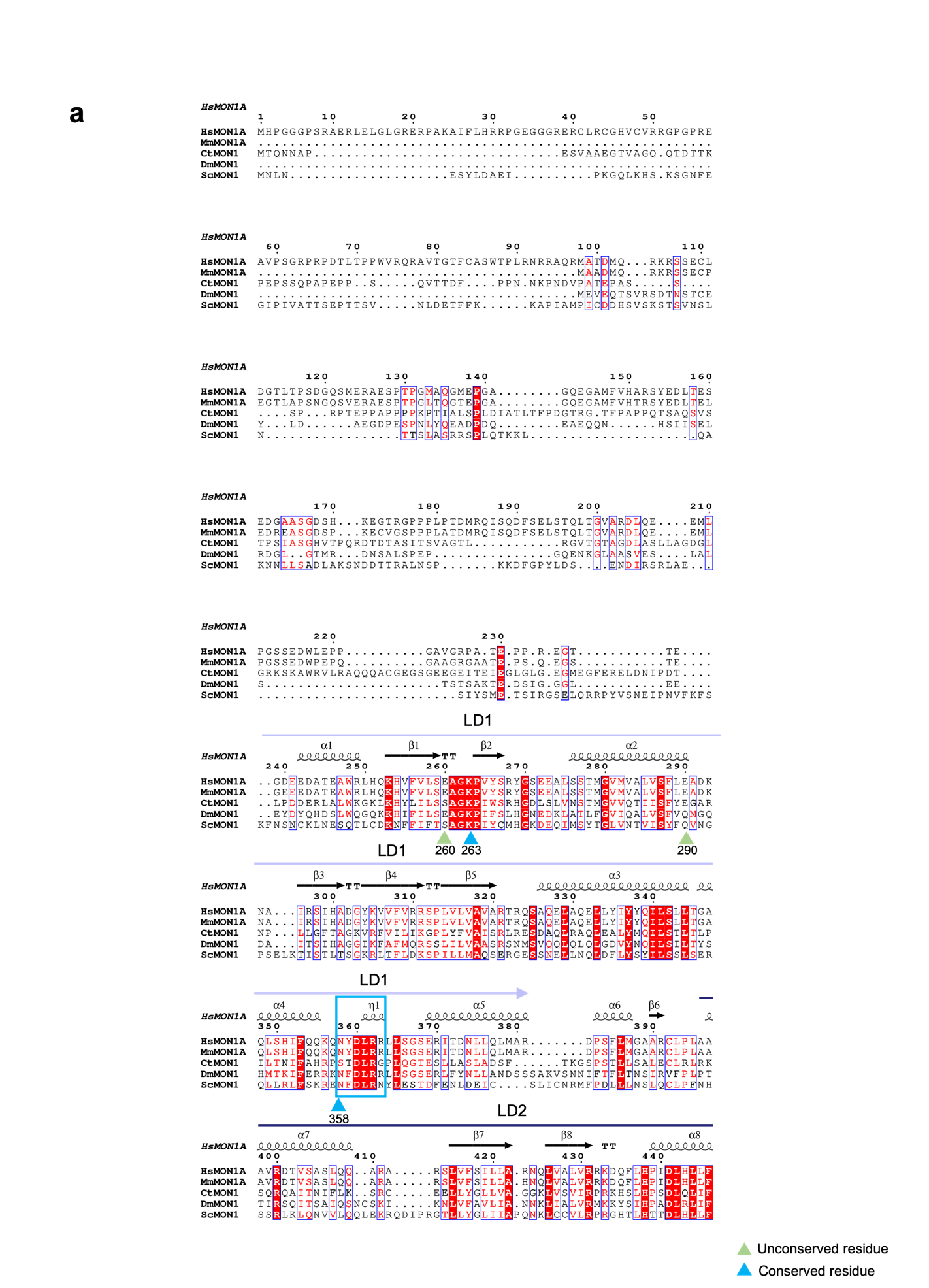


**
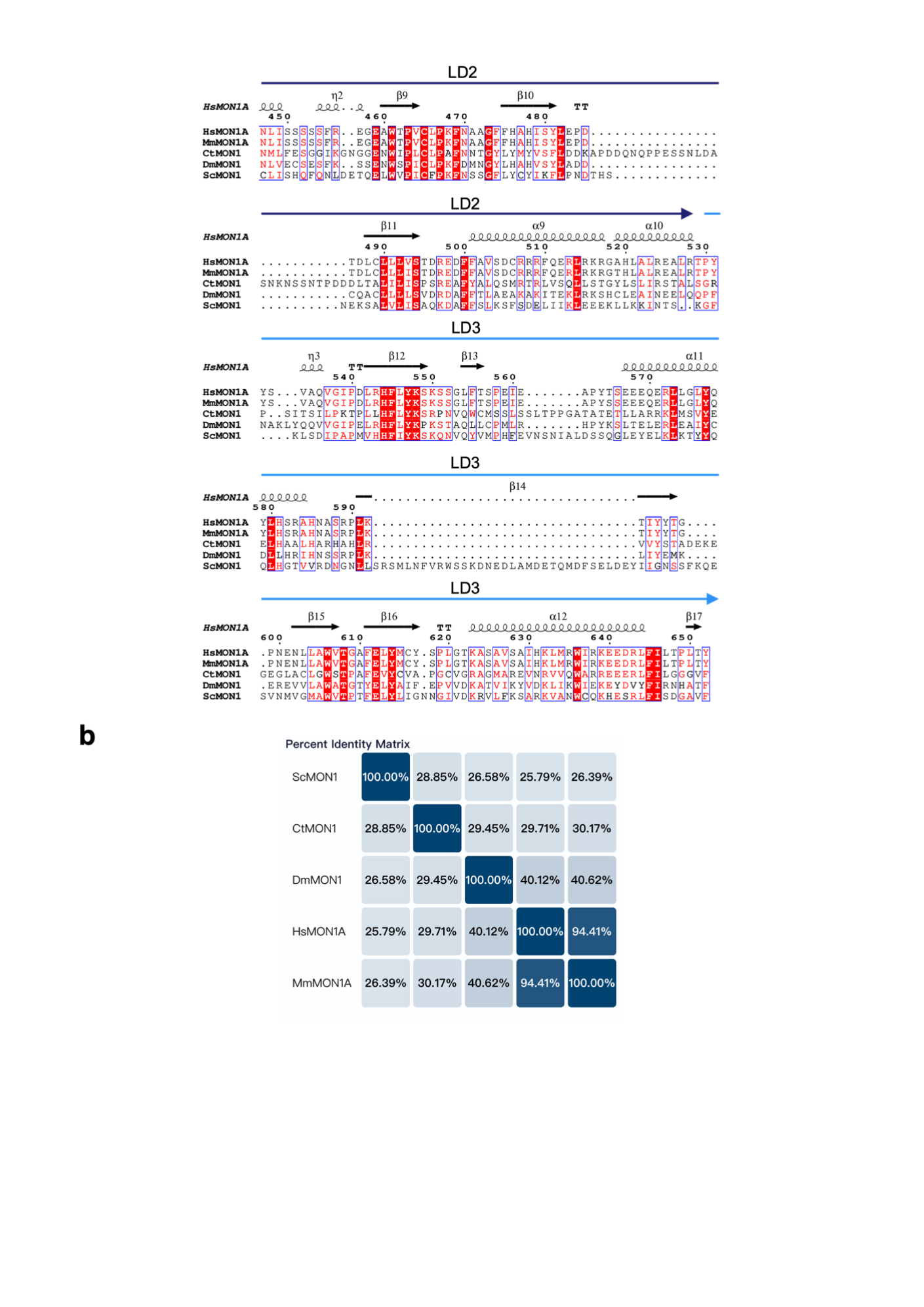
**

**Supplementary Figure S2** A multiple sequence alignment of MON1. (a) Multiple sequence alignment of MON1 proteins from Hs, *Chaetomium thermophilu* (Ct), *Drosophila melanogaster* (Dm), *Mus musculus* (Mm), and *Saccharomyces cerevisiae* (Sc), generated with ClustalW and visualized with ESPript. Similar and conserved positions are marked by red font or boxes, respectively. Secondary structure elements observed in the cryo-EM structure of HsMCR7^N125I^ are labeled above the alignment. (b) Sequence alignment of MON1 proteins from Hs, Sc, Ct, Dm, and Mm.


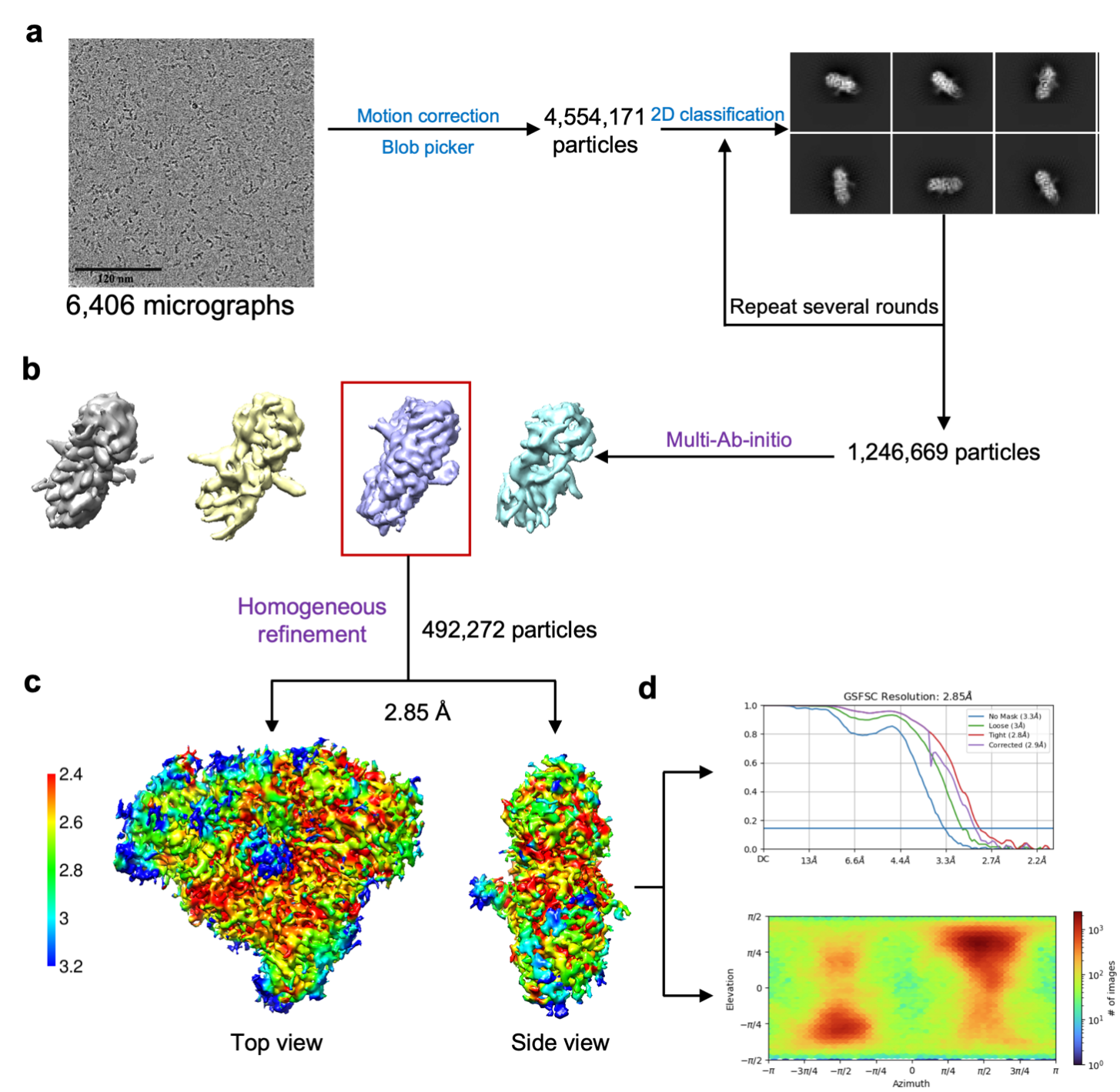


**Supplementary Figure S3** Flow chart for cryo-EM data processing and structure determination of the human MON1A-CCZ1-RAB7A^N125I^ complex. (a) Representative cryo-EM micrograph of HsMCR7 ^N125I^ and corresponding 2D class averages. (b) Workflow of classification, 3D variability, and refinement. (c) Top and side views of the final EM map are surface-rendered and color-coded by estimated local resolution. (d) Resolution estimation based on the FSC 0.143 criterion and orientation distribution map calculated using CryoSPARC. Please refer to the “Cryo-EM data processing” in the Methods for further details.


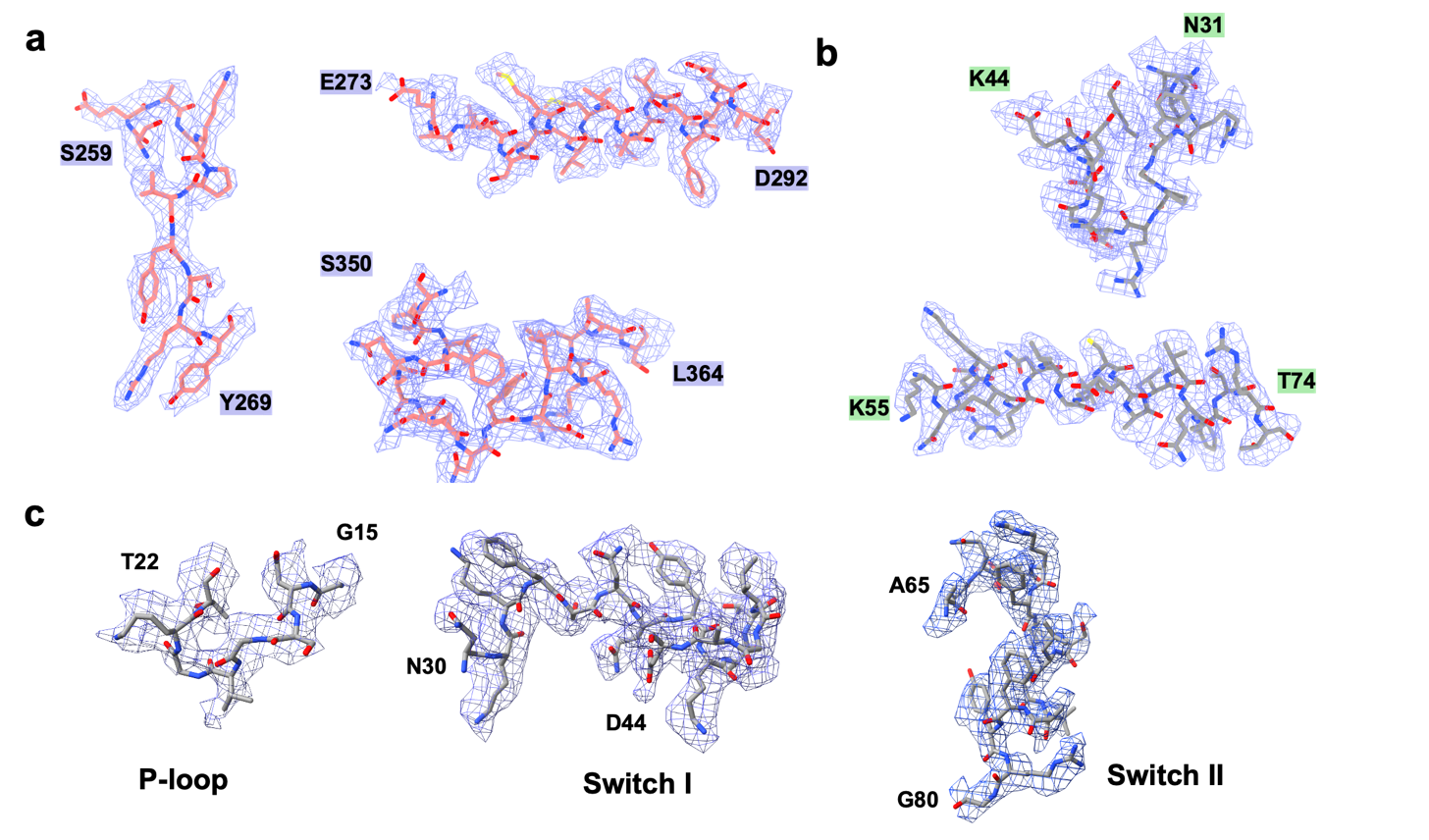


**Supplementary Figure S4** Cryo-EM map of the key residues mediating the interactions between the HsMC1 complex and RAB7A. Cryo-EM density map highlights key residues at the interface between MON1A (a), CCZ1 (b), and RAB7A (c), including critical structural elements of HsRAB7A such as the P-loop, Switch Ⅰ, and Switch Ⅱ.


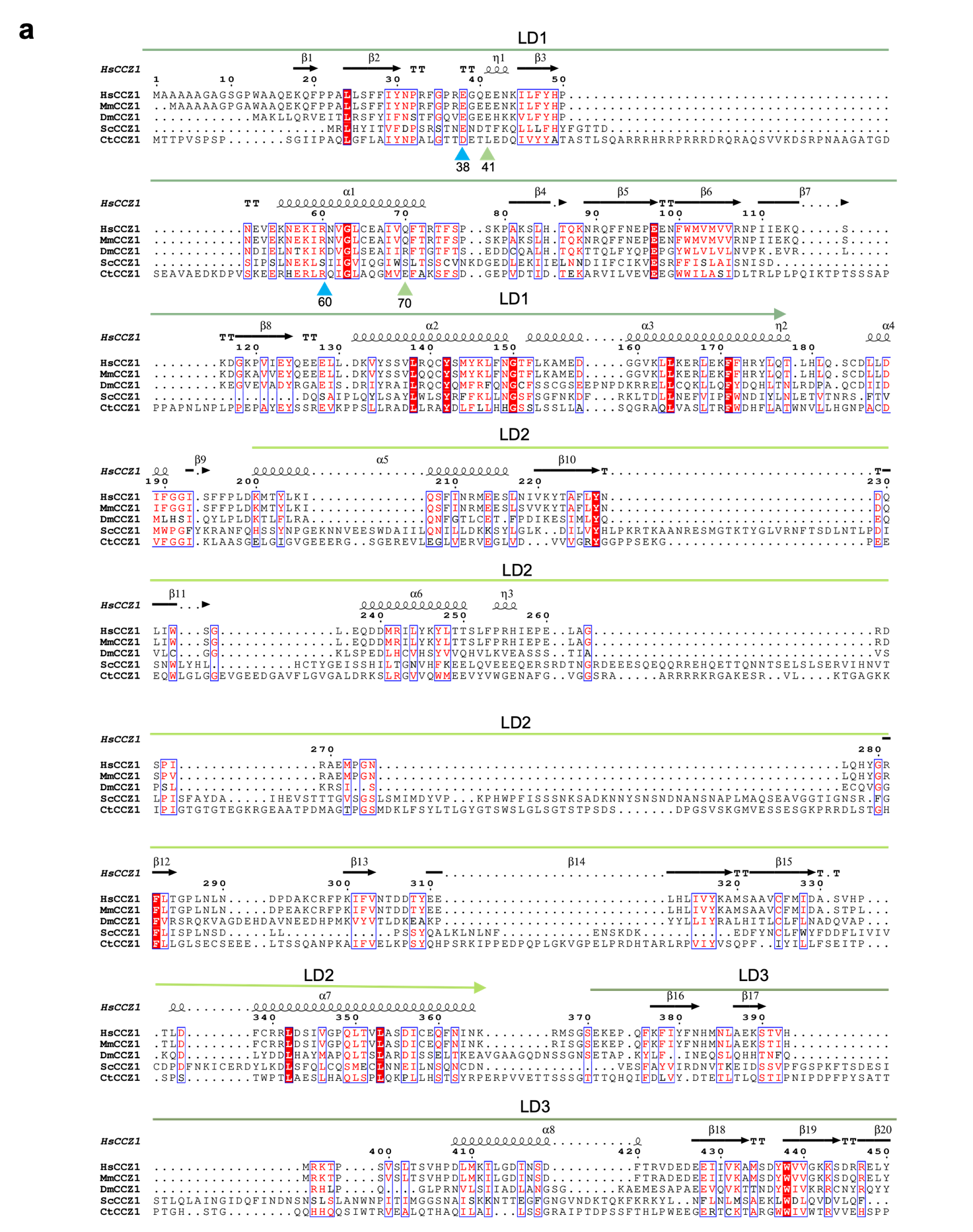


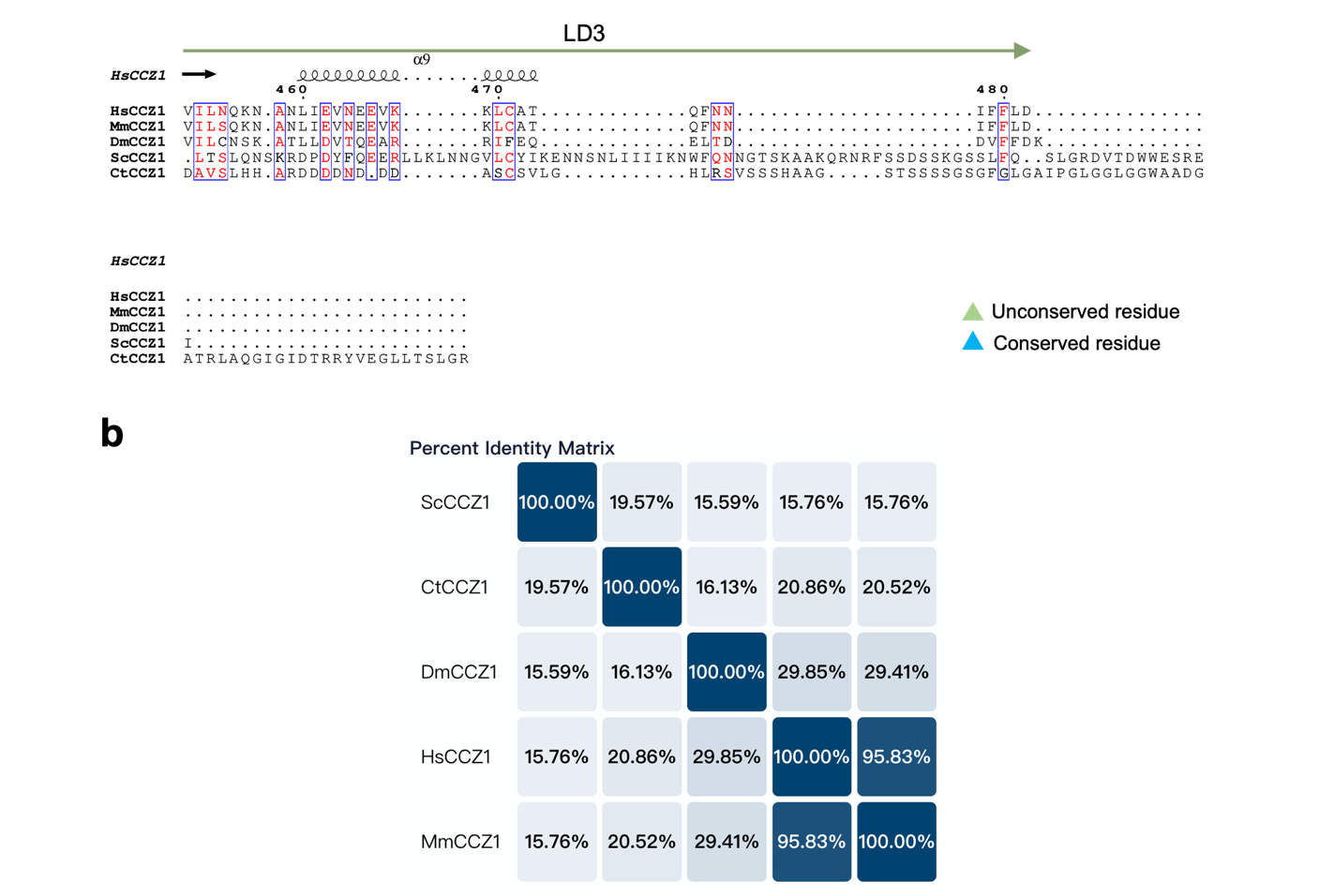


**Supplementary Figure S5** Multiple sequence alignment of CCZ1. (a) Multiple sequence alignment of CCZ1 proteins from Hs, Ct, Dm, Mm, and Sc, generated with ClustalW and visualized with ESPript. Similar and conserved positions are marked by red font or boxes, respectively. Secondary structure elements observed in the cryo-EM structure of HsMCR7^N125I^ are labeled above the alignment. (b) Sequence alignment of CCZ1 proteins from Hs, Sc, Ct, Dm, and Mm.


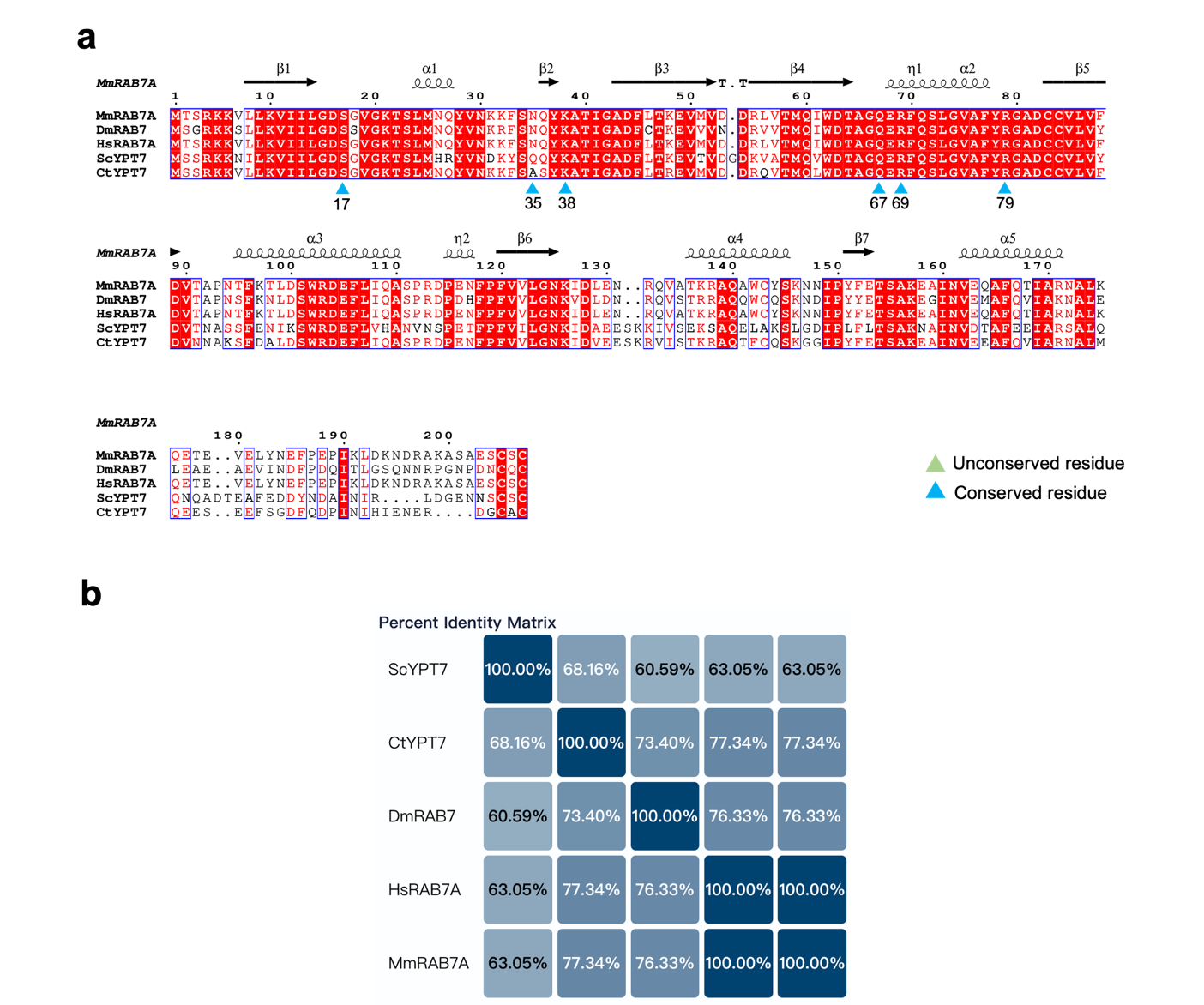


**Supplementary Figure S6** Multiple sequence alignment of RAB7A/Ypt7. (a) Multiple sequence alignment of RAB7A/Ypt7 proteins from Hs, Ct, Dm, Mm, and Sc, generated with ClustalW and visualized with ESPript. Similar and conserved positions are marked by red font or boxes, respectively. Secondary structure elements observed in the cryo-EM structure of HsMCR7^N125I^ are labeled above the alignment. (b) Sequence alignment of RAB7/Ypt7 proteins from Hs, Sc, Ct, Dm, and Mm.


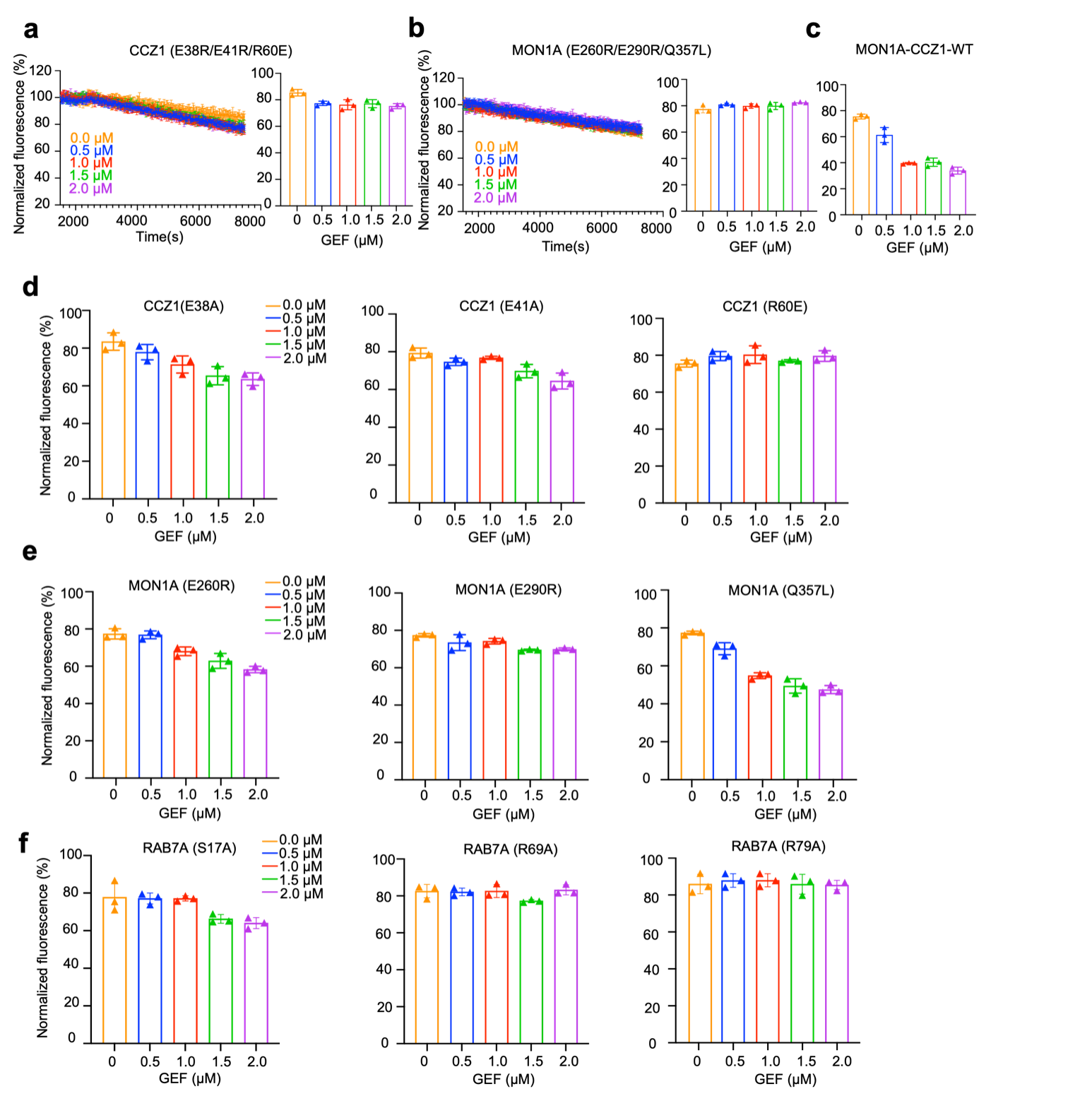


**Supplementary Figure S7** GEF assay and quantitative analysis of MON1A-CCZ1/RAB7A mutants. (a and b) MANT-GDP release monitored in the presence of RAB7A-WT and the HsMC1 triple mutants: (a) CCZ1 (E38R/E41R/R60E) and (b) MON1A (E260R/E290R/Q357L). Left: real-time fluorescence curves; right: quantification of endpoint fluorescence values. (c) Quantitative analysis of RAB7A-WT nucleotide exchange with increasing concentrations of HsMC1-WT. (d and e) Quantitative analysis of RAB7A-WT nucleotide exchange with different HsMC1 mutants bearing single-point mutations in (d) CCZ1 (E38A, E41A, and R60E) and (e) MON1A (E260R, E290R, and Q357L). (f) Quantitative analysis of RAB7A-mutants (S17A, R69A, and R79A) nucleotide exchange with increasing concentrations of the HsMC1-WT complex. All quantitative data represent mean ± SD from three independent experiments and correspond to fluorescence values at 7200 s.


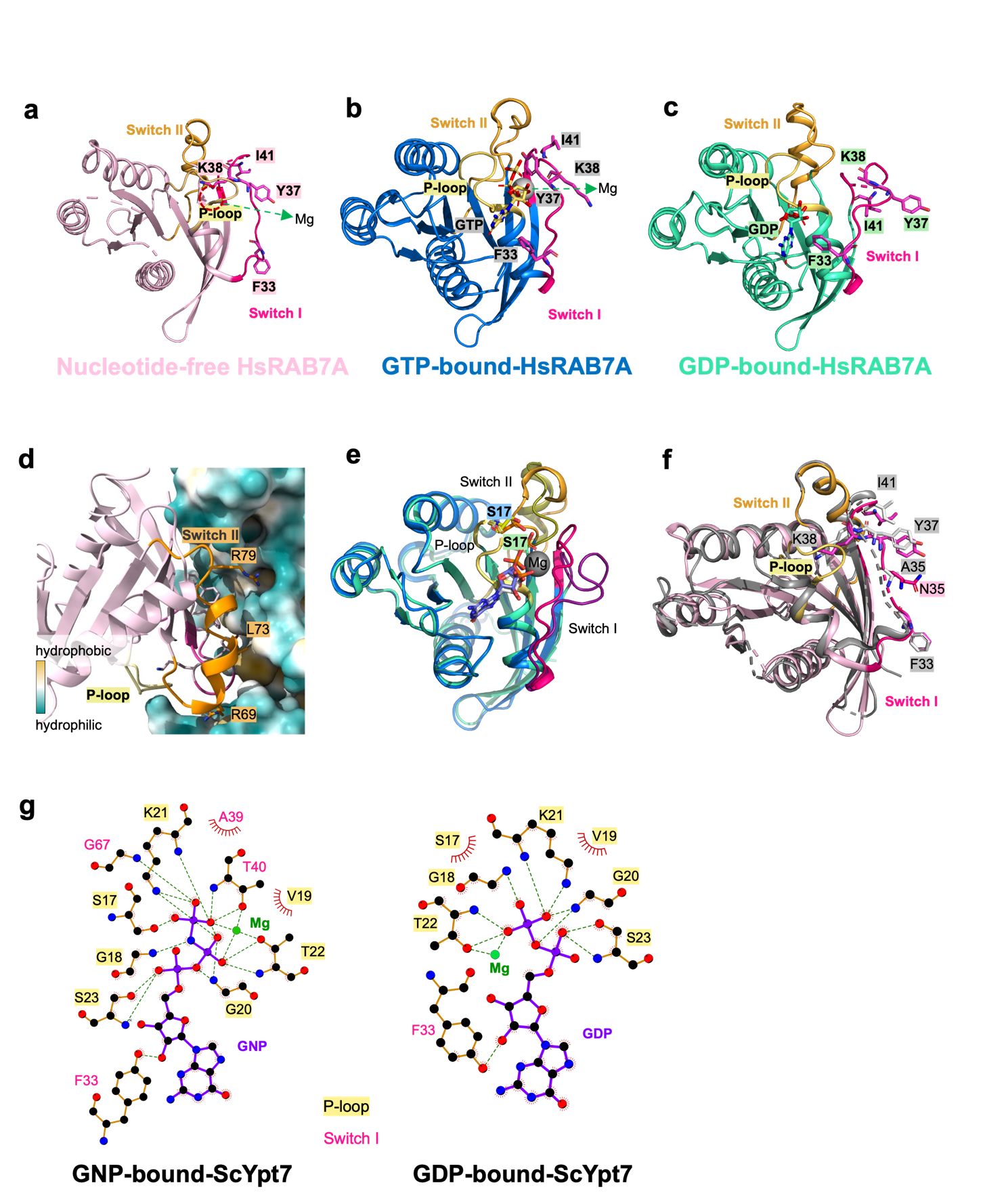


**Supplementary Figure S8** Comparison of different conformations of RAB7A/Ypt7. (a−c) Cartoon representation showing the structure of nucleotide-free-HsRAB7A (colored in light pink) (a), GTP-bound-HsRAB7A (PDB: 1T91, colored in blue) (b) and GDP-bound-HsRAB7A in the ORP1L-RAB7A-Q67L complex (PDB:8ZQ3, colored in green) (c). The location of the proposed Mg^2+^ is indicated by a red dashed line. The Switch Ⅰ, Switch Ⅱ, and P-loop regions are colored hot pink, yellow-orange, and pale yellow, respectively. Residues F33, Y37, K38, and I41 are displayed as sticks and labeled. (d) Interaction of R69, L73, and R79 from Switch Ⅱ of HsRAB7A with hydrophobic pockets on the HsMC1 complex. The surface of the HsMC1 complex is colored according to hydrophobicity from green (hydrophilic) to yellow (hydrophobic). (e) Superposition of GTP-bound HsRAB7A and GDP-bound-HsRAB7A, with the S17 residue in the P-loop indicated. (f) Superposition of empty form of HsRAB7A and CtYpt7 (colored in grey), with key residues (F33, A35, N35, Y37, K38, and I41) labeled and shown as sticks. (g) Schematic diagram of interactions formed between GNP and the ScYpt7 (PDB: 1KY2) and between GDP and ScYpt7 (PDB: 1KY3).


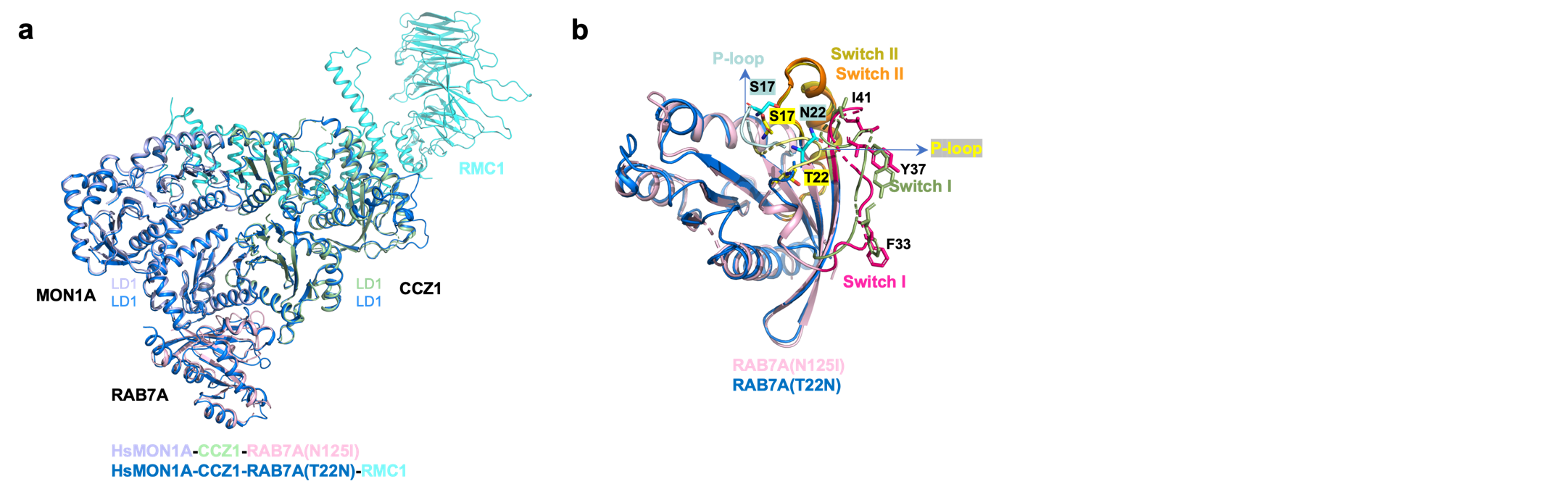


**Supplementary Figure S9** Comparison of the structures of human MON1A-CCZ1-RAB7A ^N125I^ and MON1A-CCZ1-RAB7A^T22N^-RMC1 complexes. (a) Superposition of human MON1A-CCZ1-RAB7A^N125I^ and MON1A-CCZ1-RAB7A^T22N^-RMC1 (PDB: 9LOD). The MON1A, CCZ1, and RAB7A of human MON1A-CCZ1-RAB7A^N125I^ are colored in light blue, pale green, and light pink, respectively. The MON1A-CCZ1- RAB7A^T22N^ and RMC1 are colored in marine blue and cyan, respectively. (b) Superposition of human RAB7A^N125I^ and RAB7A^T22N^ from their complexes in Supplementary Figure S9a, respectively. The Switch Ⅰ, Switch Ⅱ, and P-loop regions of HsRAB7A^N125I^ are colored hot pink, olive orange, and yellow, respectively. The Switch Ⅰ, Switch Ⅱ, and P-loop regions of HsRAB7A^T22N^ are colored smudge green, orange, and palecyan, respectively. Residues T22, S17, F33, Y37, I41, and N22 are displayed as sticks and labeled.

**Supplementary Table S1** Cryo-EM data collection, refinement, and validation statistics.

| **Dataset** | **Human MON1A-CCZ1-RAB7A^N125I^** | |
| --- | --- | --- |
| **Data collection and processing** |  |  |
| Magnification | 75,000 | |
| Voltage (kV) | | 300 |
| Electron exposure (e^−^/Å^2^) | | 50 |
| Defocus range (μm) | | −1.5 − −2.5 |
| Pixel size (Å) | | 1.036 |
| Micrographs (no.) | | 6,406 |
| Symmetry imposed | | C1 |
| Final particle images (no.) | | 492,272 |
| Map resolution (Å) 0.143 FSC threshold | | 2.85 |
| Map sharpening B factor (Å^2^) | | −130.4 |
| **Refinement** | |  |
| Model composition | |  |
| Chain count | | 3 |
| Non-hydrogen atoms | | 8,084 |
| Protein residues | | 993 |
| Ligands | | 0 |
| B factors (Å^2^) | |  |
| Proteins | | 40.90 |
| Nucleotide | | --- |
| R.m.s deviations | |  |
| Bond lengths (Å) | | 0.004 |
| Bond angles (°) | | 0.591 |
| Validation | |  |
| MolProbity score | | 2.24 |
| Clash score | | 10.02 |
| Poor rotamers (%) | | 3.84 |
| Ramachandran plot | |  |
| Favored (%) | | 96 |
| Allowed (%) | | 3.69 |
| Outlier (%) | | 0.31 |
| PDB code | | 9LOL |
| EMDB code | | EMD-63249 |
